# Supplementary figures and images for: Ultrafast photochemical processes in 1,2-dichloroethene measured with a universal XUV probe
Source: Phys Chem Chem Phys. 2024 Oct 30;26(45):28406–16. doi: 10.1039/d4cp02952f (PMC11563349; doi:10.1039/d4cp02952f)

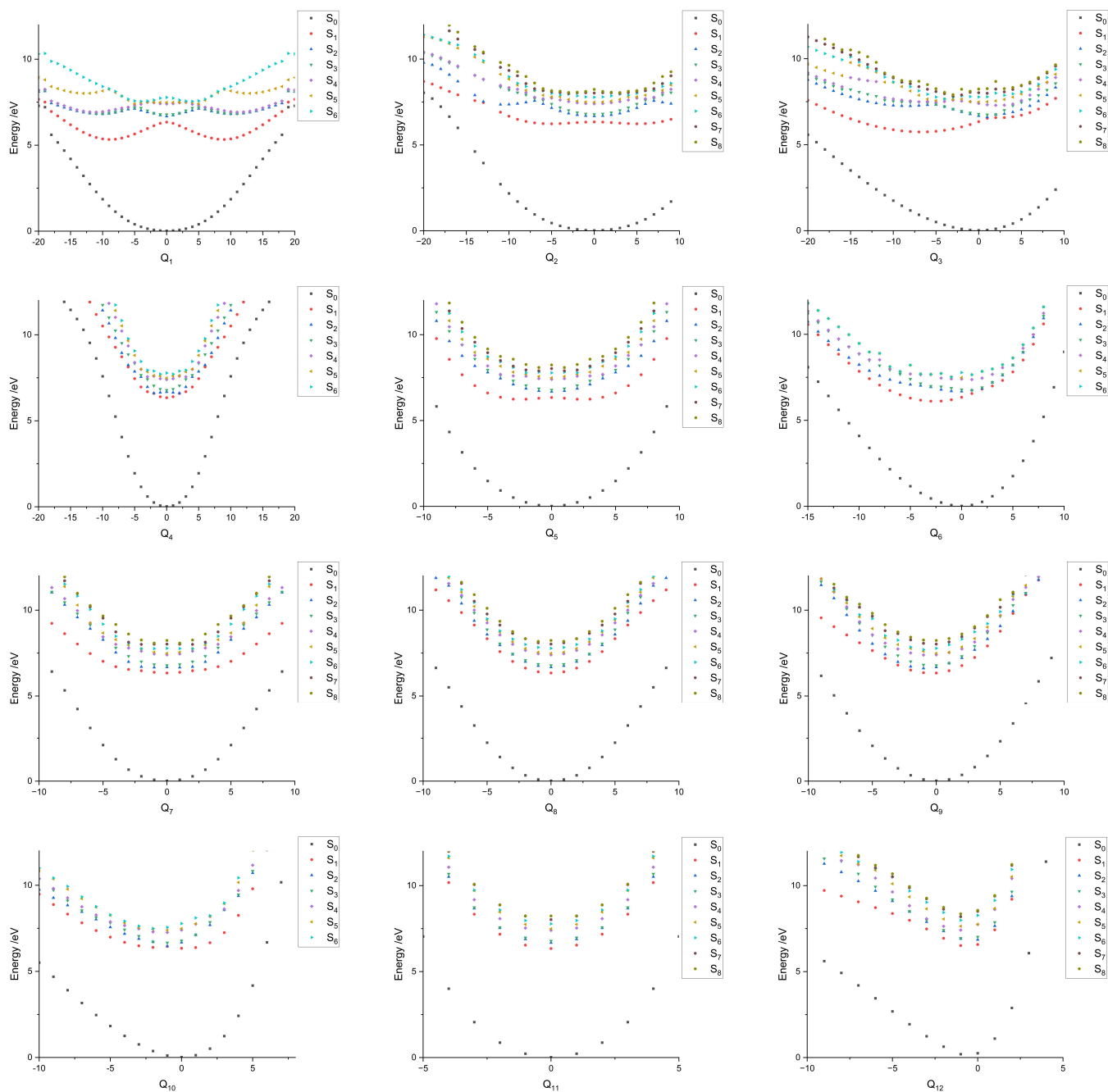

Supplement: CP-026-D4CP02952F-s001 [file CP-026-D4CP02952F-s001.pdf]

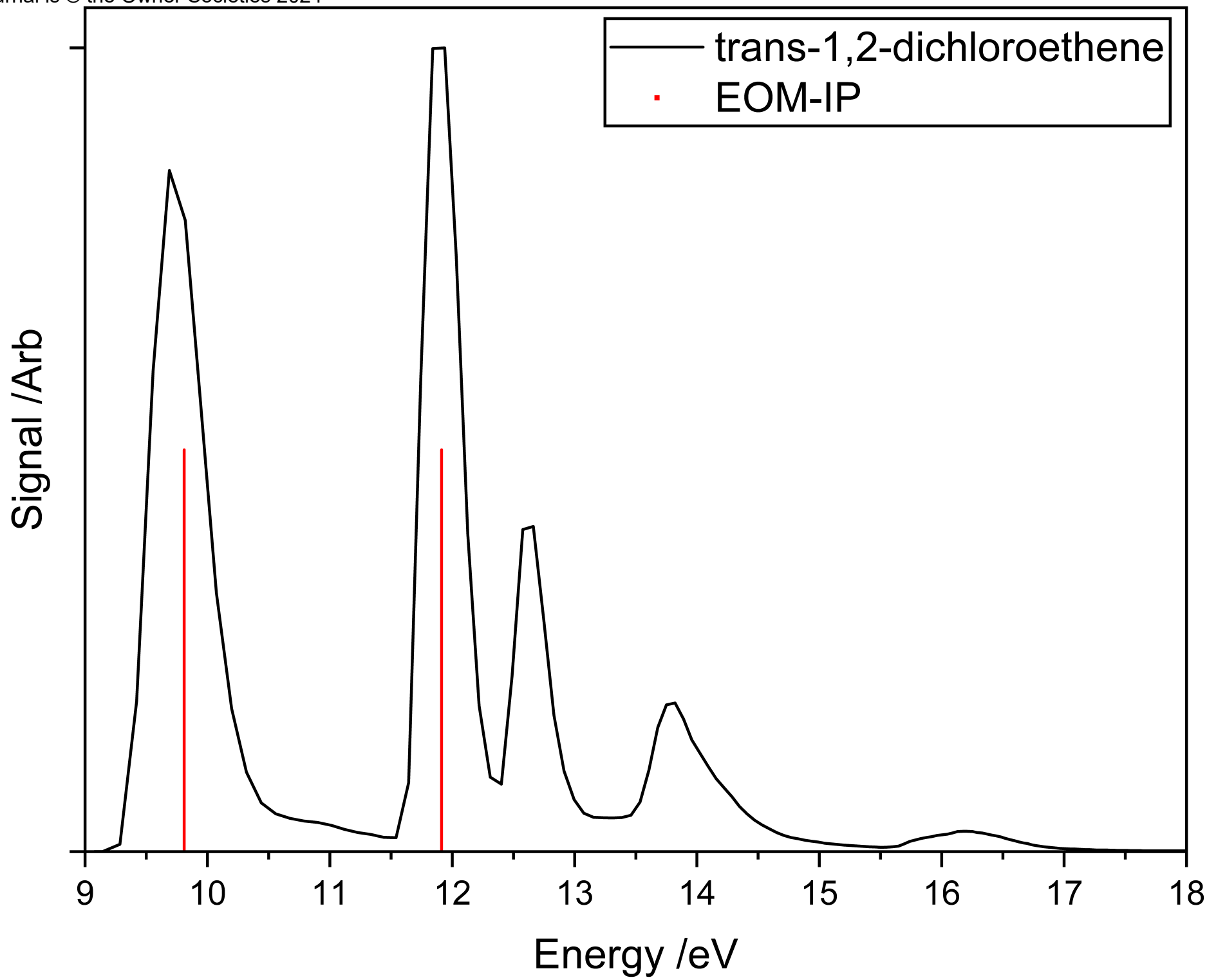

Supplement: CP-026-D4CP02952F-s003 [file CP-026-D4CP02952F-s003.pdf]

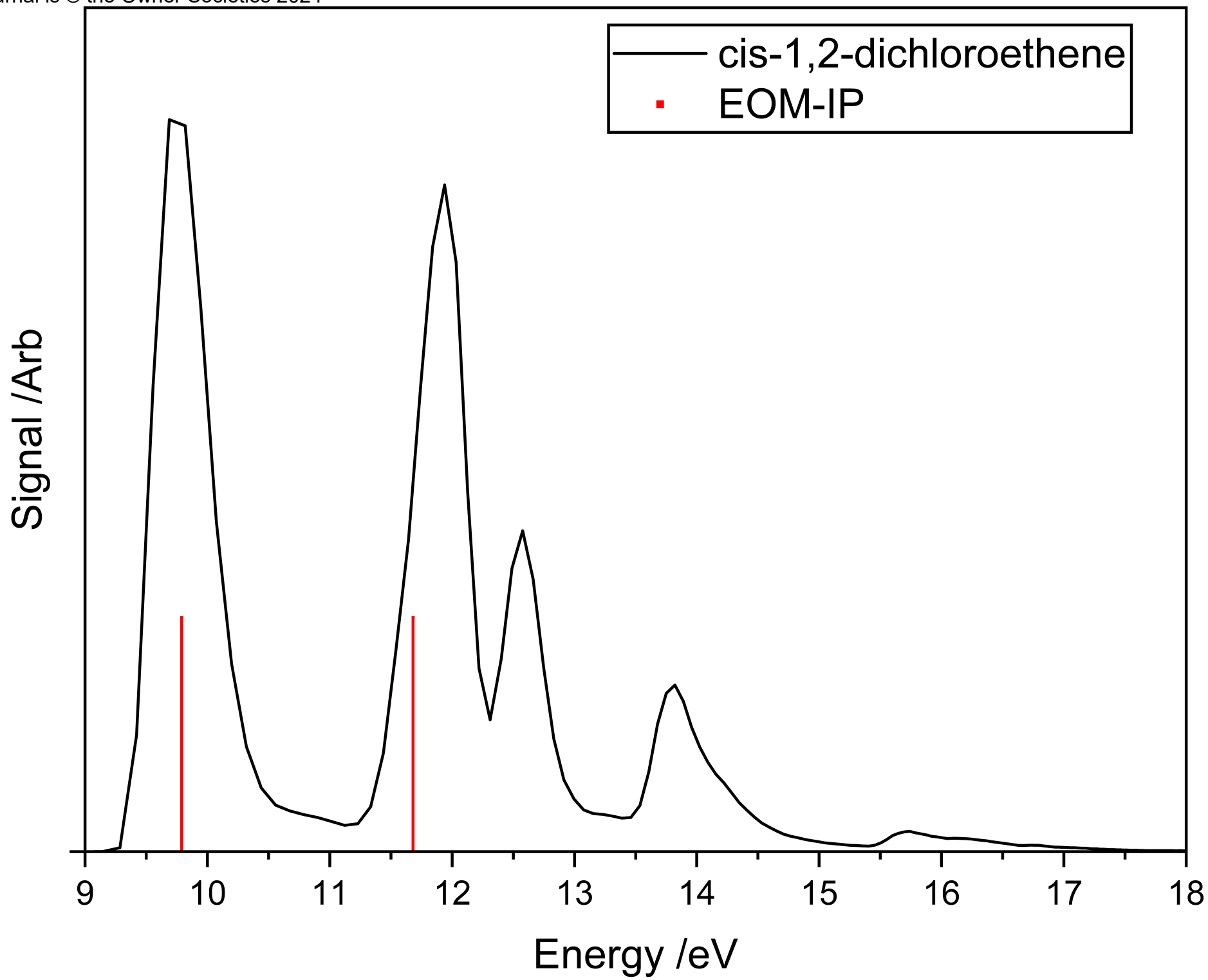

Supplement: CP-026-D4CP02952F-s004 [file CP-026-D4CP02952F-s004.pdf]

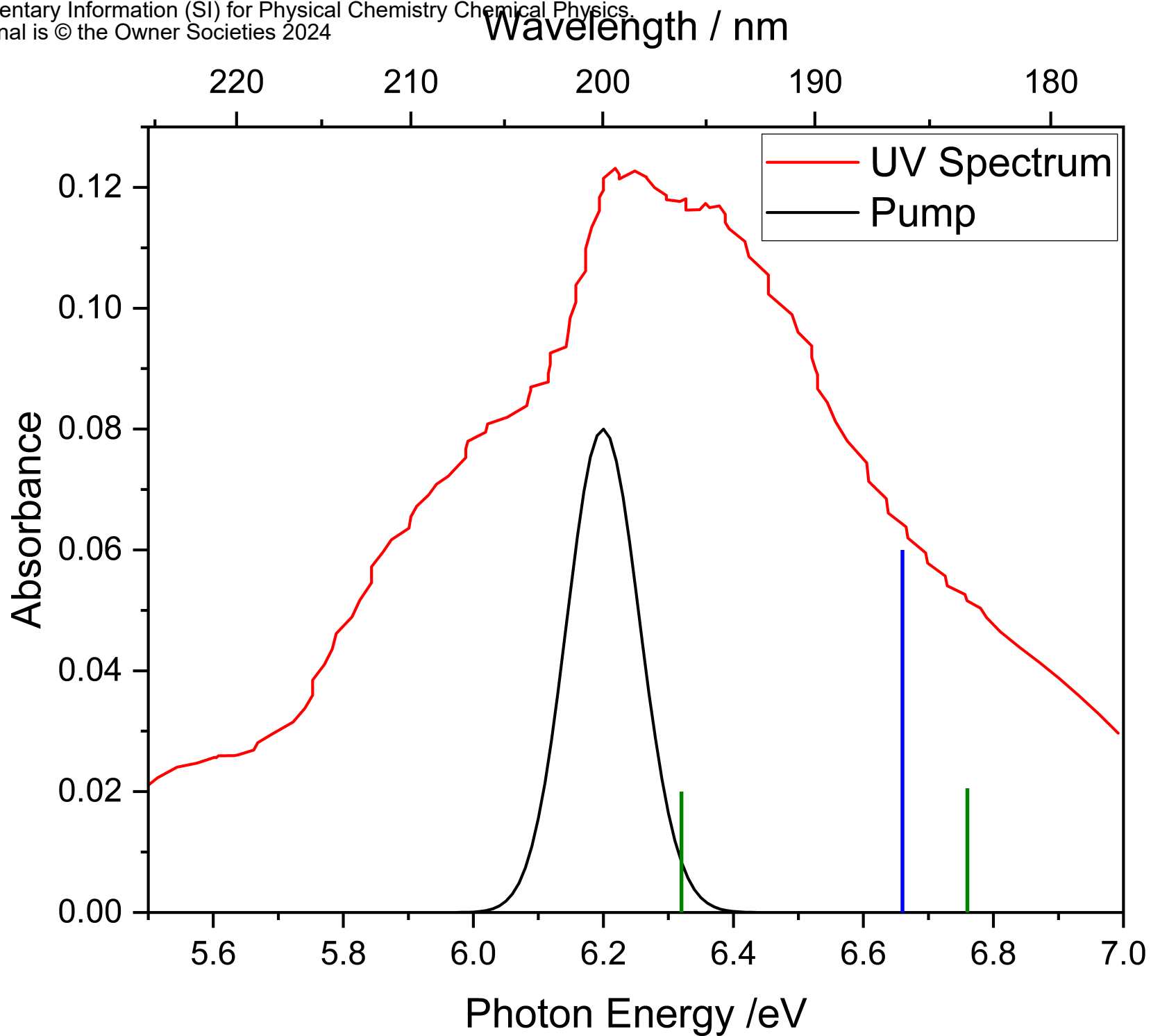

Supplement: CP-026-D4CP02952F-s005 [file CP-026-D4CP02952F-s005.pdf]

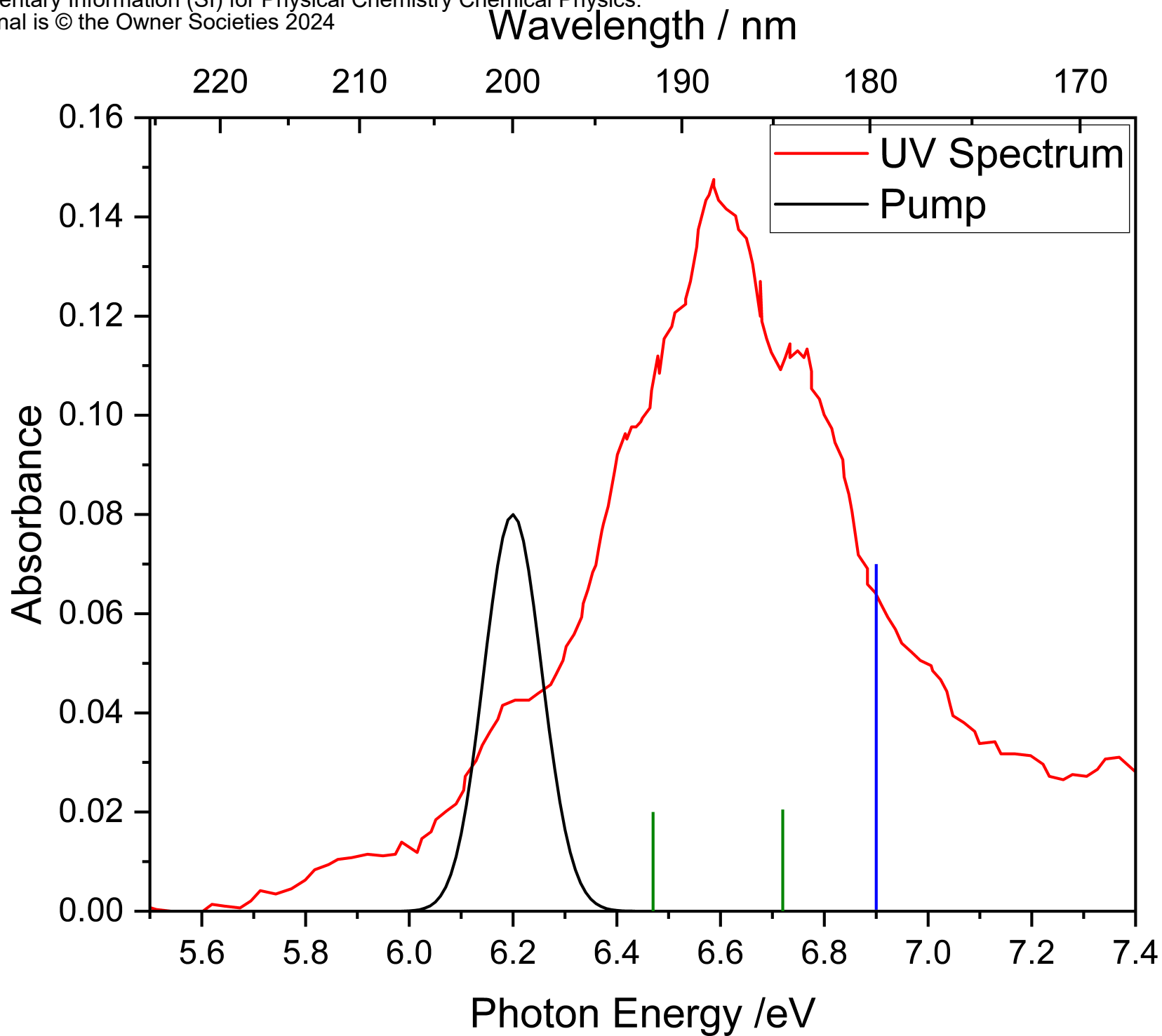

Supplement: CP-026-D4CP02952F-s006 [file CP-026-D4CP02952F-s006.pdf]

Norm. intensity / arb. units

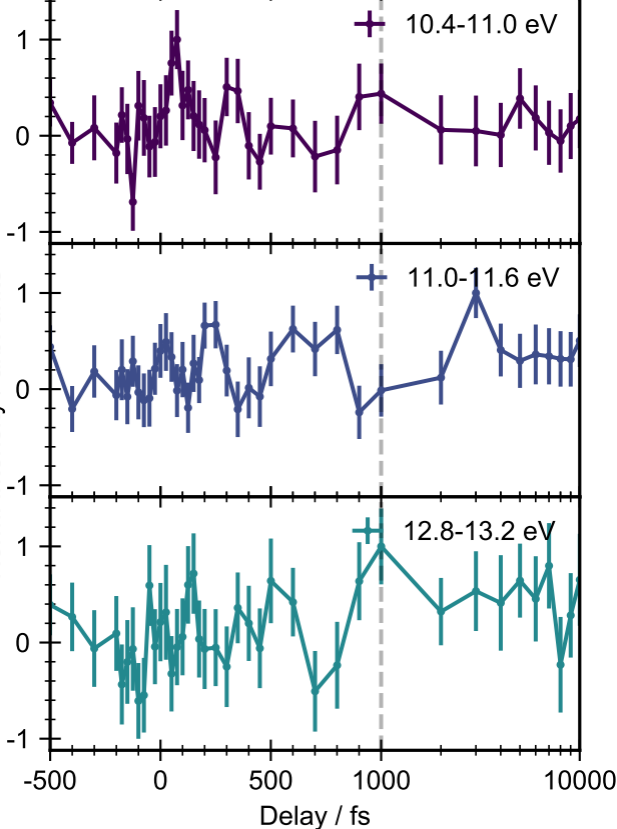

Supplement: CP-026-D4CP02952F-s007 [file CP-026-D4CP02952F-s007.pdf]

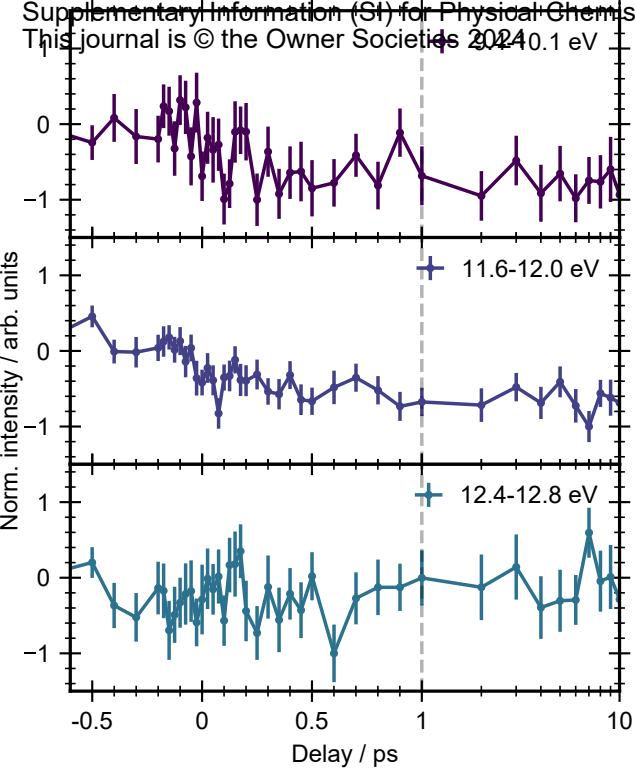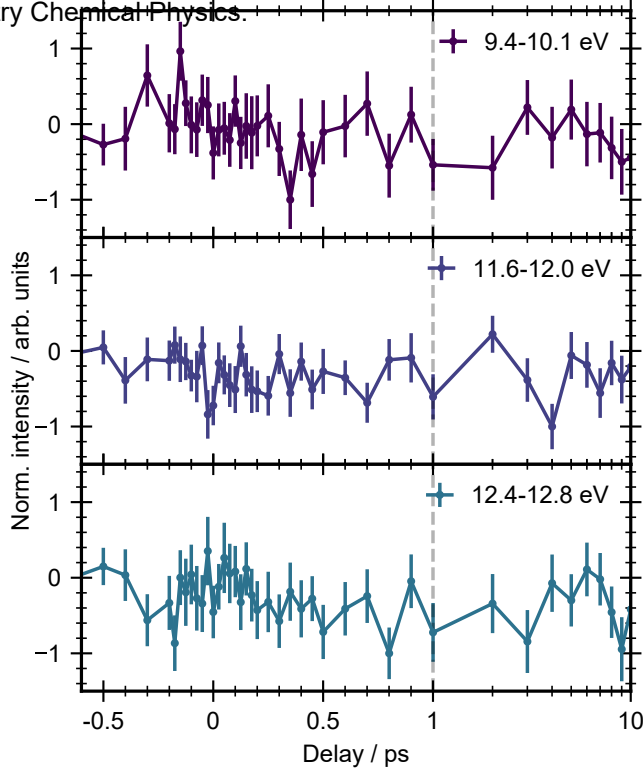

Supplement: CP-026-D4CP02952F-s008 [file CP-026-D4CP02952F-s008.pdf]

Norm. intensity / arb. units

12.8-13.2 eV

-0.5

0

0.5

1

10

Delay / ps

$$\tau_1 = 18 \pm 5 \text{ fs}$$

$$\tau_2 = 3075 \pm 2809 \text{ fs}$$

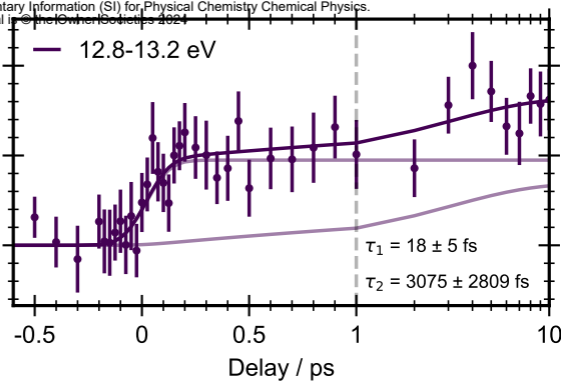

Supplement: CP-026-D4CP02952F-s009 [file CP-026-D4CP02952F-s009.pdf]

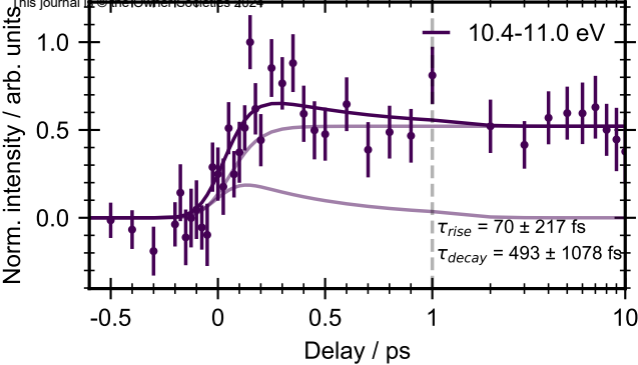

Supplement: CP-026-D4CP02952F-s010 [file CP-026-D4CP02952F-s010.pdf]
