# Supplementary material for: Ultrafast photochemical processes in 1,2-dichloroethene measured with a universal XUV probe
Source: Phys Chem Chem Phys. 2024 Oct 30;26(45):28406–16. doi: 10.1039/d4cp02952f (PMC11563349; doi:10.1039/d4cp02952f)
Supplement: CP-026-D4CP02952F-s002 [file CP-026-D4CP02952F-s002.pdf]

Supplementary Information (SI) for Physical Chemistry Chemical Physics.
This journal is © the Owner Societies 2024

Supplementary Information

Henry G. McGhee, Henry J. Thompson, James Thompson,
Yu Zhang, Adam S. Wyatt, Emma Springate, Richard T. Chapman,
Rebecca A. Ingle, Daniel A. Horke, Russell S. Minns, Michael A. Parkes

Department of Chemistry, University College London, 20 Gordon Street, London, WC1H 0AJ, UK

E-mail: michael.parkes@ucl.ac.uk

1 Calculated Geometry

Atom	X / Bohr	Y / Bohr	Z / Bohr
C	0.493660842	0.442675934	0
H	0.334309043	1.509306884	0
C	-0.493660842	-0.442675934	0
H	-0.334309043	-1.509306884	0
Cl	-2.144621671	0.048817983	0
Cl	2.144621671	-0.048817983	0

Table S1: Optimised structure for *trans*-1,2-dichloroethene from a CCSD calculation with a 6-311G++(3df, 3pd) basis.

Atom	X / Bohr	Y / Bohr	Z / Bohr
C	0	0	0
H	0	0	1.0792497
C	1.1533616	0	-0.6696234
Cl	-1.5402877	0	-0.7320455
Cl	1.2812994	0	-2.3702141
H	2.0906222	0	-0.1345322

Table S2: Optimised structure for *cis*-1,2-dichloroethene from a CCSD calculation with a 6-311G++(3df, 3pd) basis.

2 Vibrational Analysis

Symmetry	Calc / cm^{-1}	NIST / cm^{-1}	Period / fs	Character
1 A_u	216.15	227	147	Torsion
2 B_u	243.09	250	133	CCCl deform
3 A_g	361.43	350	95	CCCl deform
4 B_g	798.56	763	44	CH bend
5 B_u	861.23	828	40	CCl str
6 A_g	886.05	846	39	CCl str
7 A_u	942.87	900	37	CH bend
8 B_u	1242.95	1200	28	CH bend
9 A_g	1318.17	1274	26	CH bend
10 A_g	1677.01	1578	21	CC str
11 B_u	3251.41	3073	11	CH str
12 A_g	3253.76	3090	11	CH str

Table S3: Calculated Frequencies for *trans*-1,2-dichloroethene from a CCSD calculation with a 6-311G++(3df, 3pd) basis. Experimental values taken from NIST webbook[1]

Symmetry	Calc / cm^{-1}	NIST / cm^{-1}	Period / fs	Character
1 A_1	172.56	173	193	CCCl deform
2 A_2	421.04	406	82	Torsion
3 B_1	589.81	571	58	CCCl deform
4 B_2	720.82	697	48	CH bend
5 A_1	760.19	711	47	CCl str
6 B_1	902.92	857	39	CCl str
7 A_2	905.75	876	38	CH bend
8 A_1	1233.8	1179	28	CH bend
9 B_1	1343.76	1303	25	CH bend
10 A_1	1662.24	1587	21	CC str
11 B_1	3229.88	3072	11	CH str
12 A_1	3252.34	3077	11	CH str

Table S4: Calculated Frequencies for *cis*-1,2-dichloroethene from a CCSD calculation with a 6-311G++(3df, 3pd) basis. Experimental values taken from NIST webbook[1]

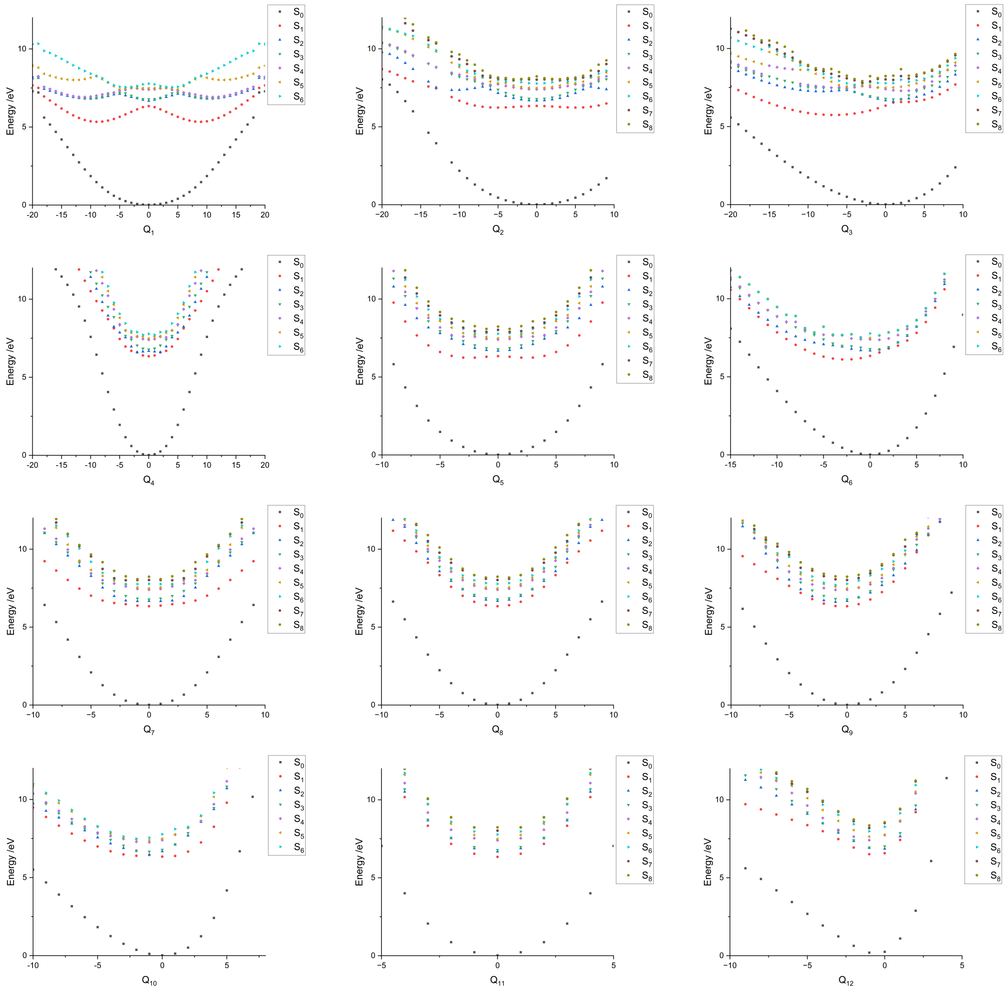

Figure S1: Cuts through the potential energy surface of *trans*-1,2-dichloroethene along mass-weighted normal modes. Calculated using EOM-CCSD/6-311G++(3df,3pd) in QChem. The number of the mode is indicated in the x-axis label.

3 Ionization Energies

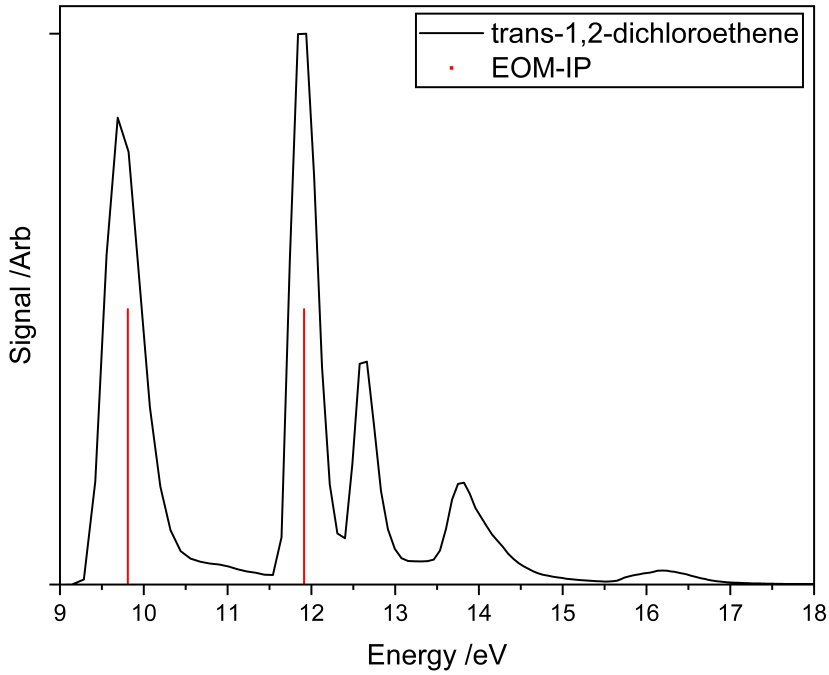

Figure S2: Comparison of our *trans*-1,2-dichloroethene XUV photoelectron spectrum at 21.64 eV with calculated ionization energies using EOM-CCSD with a 6-311G++(3df, 3pd) basis set

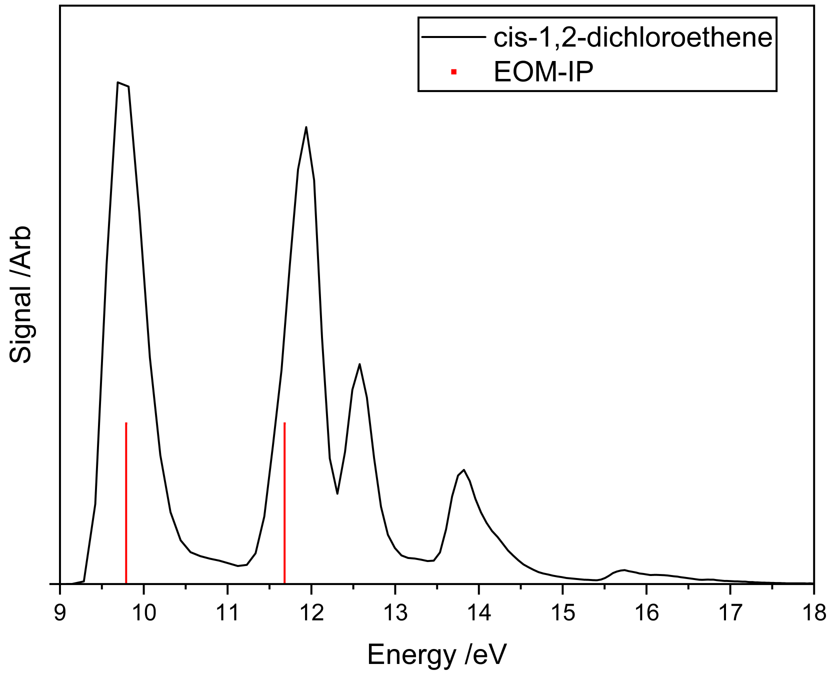

Figure S3: Comparison of our *cis*-1,2-dichloroethene XUV photoelectron spectrum at 21.64 eV with calculated ionization energies using EOM-CCSD with a 6-311G++(3df, 3pd) basis set

4 Photoabsorption

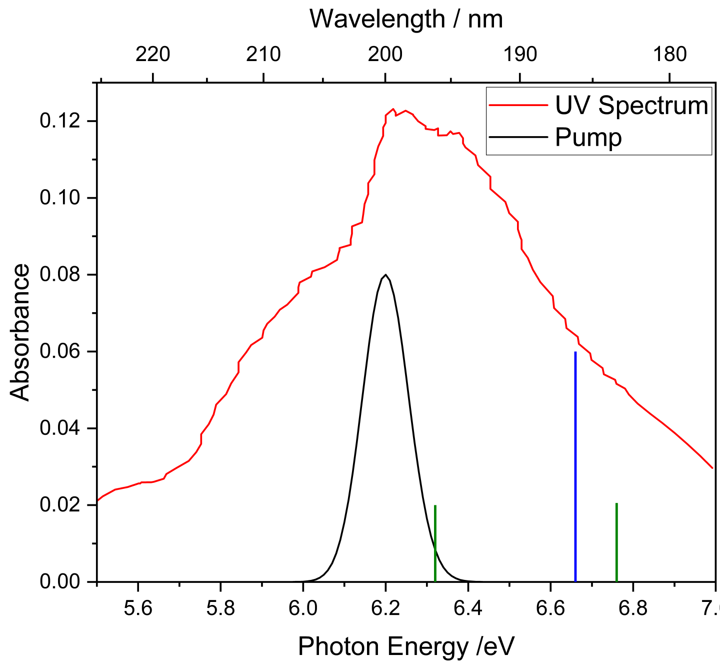

Figure S4: UV-vis spectrum of *trans*-1,2-dichloroethene taken from Locht *et al*,[2] shown with position of pump pulse (black Gaussian) and the calculated positions of the first three excited states from EOM-CCSD calculations. The green lines are for states with no or very low oscillator strengths

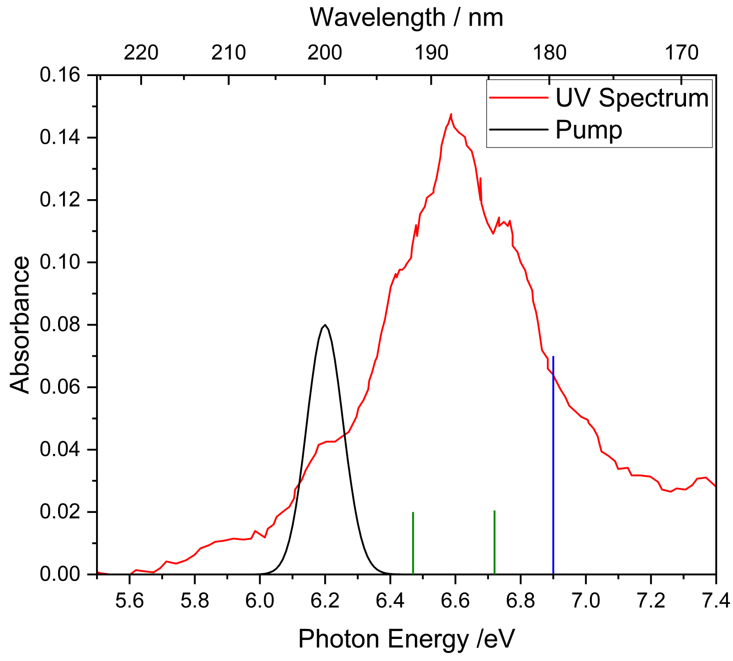

Figure S5: UV-vis spectrum of *cis*-1,2-dichloroethene taken from Locht *et al*,[3] shown with position of pump pulse (black Gaussian) and the calculated positions of the first three excited states from EOM-CCSD calculations. The green lines are for states with no or very low oscillator strengths

5 *Cis*-1,2-dichloroethene time resolved photoelectron spectroscopy results

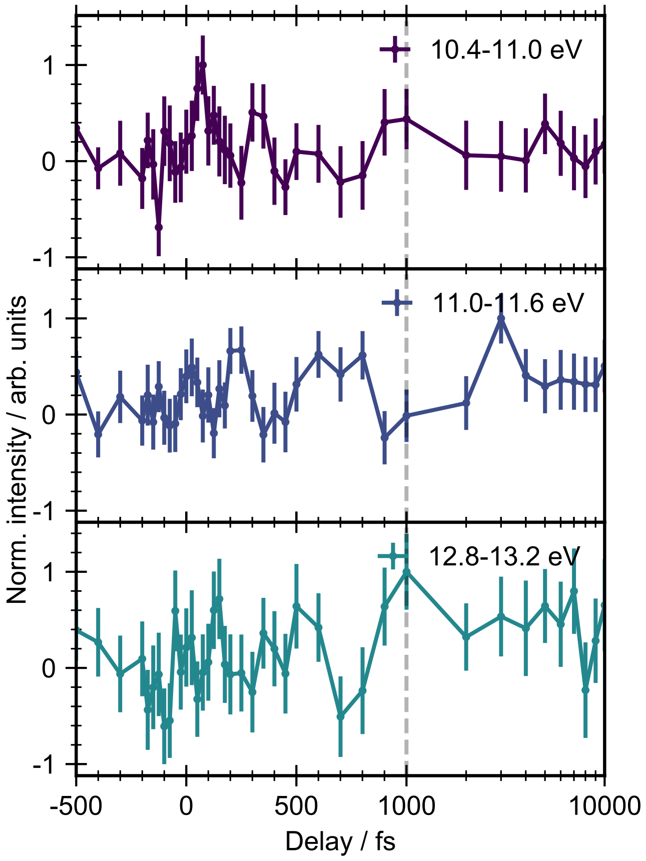

Figure S6: Integrated kinetic traces for the expected photoproduct regions (10.4-11.0, 11.0-11.6 eV and 12.8-13.2 eV) for *cis*-1,2-dichloroethene. No signal is observed above the noise level.

6 Bleach Signals

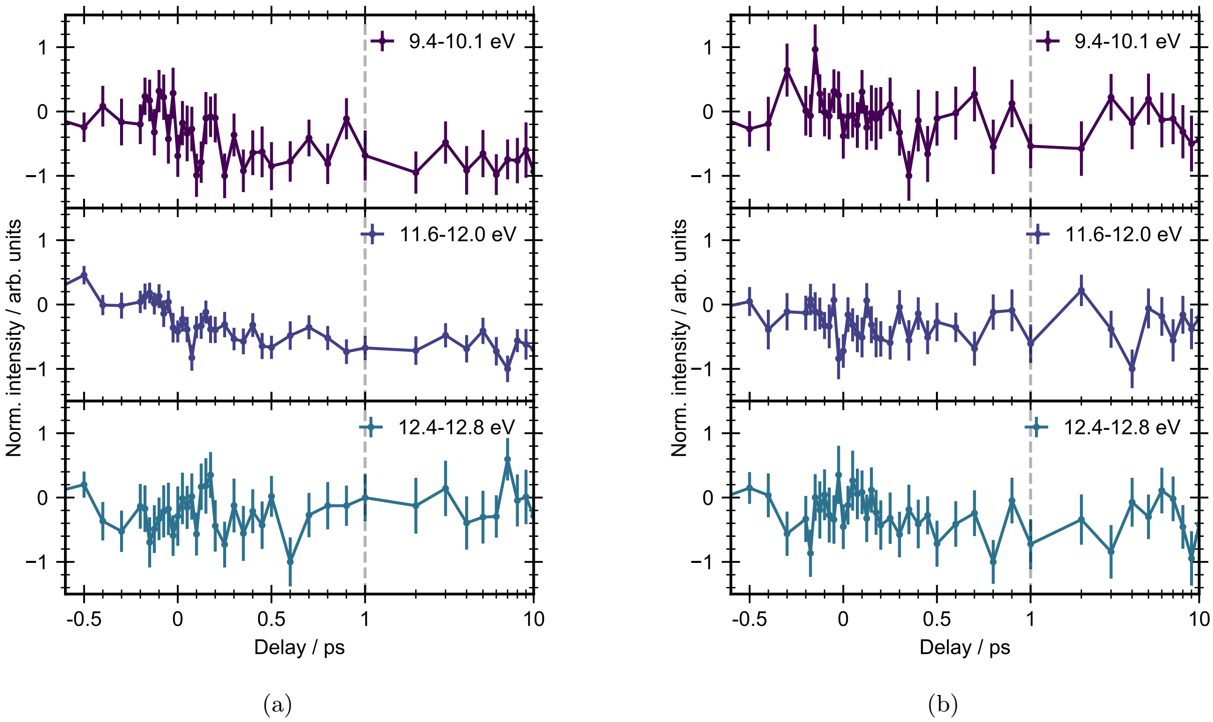

Figure S7: Integrated kinetic traces of the ground state bleach features for the first (9.4-10.1 eV), second (11.6-12.0 eV) and third (12.4-12.8 eV) ionisation potentials for (a) *trans*-1,2-dichloroethene and (b) *cis*-1,2-dichloroethene. The delay axis is plotted on a log scale for values greater than 1 ps.

7 Cl region

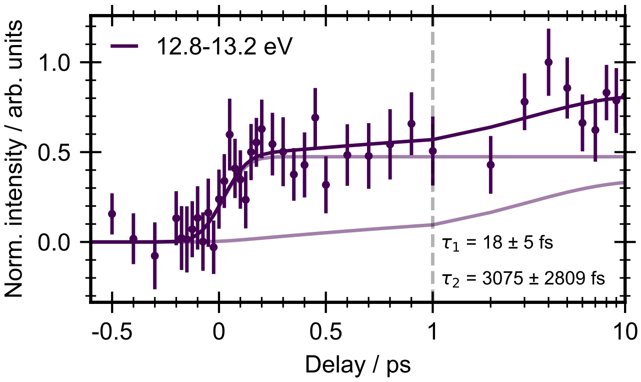

Figure S8: Integrated kinetic trace of the Cl region (12.8-13.2 eV) showing the fit with the sum of two exponential rises (convoluted with the Gaussian IRF). Individual fit components are shown by faint lines and the sum is shown by the bold line. The longer time constant may be concurrent with the slow decay (∼2 ps) of the 8.8-9.2 eV region, however the errors on the fit are large and so no definitive assignments can be made.

8 C_2HCl region

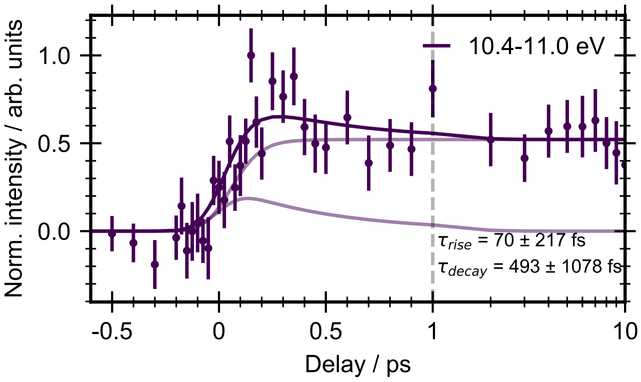

Figure S9: Integrated kinetic trace for the C_2HCl region (10.4-11.0 eV) showing the fit with an exponential decay and an exponential rise in order to account for the peak in signal at around 0.3 ps. While this qualitatively produces a better fit, the errors from the least-squares fitting process are very large and so no definitive assignments can be made.

References

[1] *NIST Chemistry WebBook, NIST Standard Reference Database Number 69*, ed. P. J. Linstrom and W. G. Mallard, National Institute of Standards and Technology, Gaithersburg MD, 20899, retrieved 2021.

[2] R. Locht, D. Dehareng and B. Leyh, *J. Quant. Spec. Rad. Trans.*, 2020, **251**, 107048.

[3] R. Locht, D. Dehareng and B. Leyh, *AIP Adv.*, 2019, **9**, 015305.
